# Comparison of microcurrent and low level laser therapy on matrix metalloproteinases and tissue inhibitors of metalloproteinases expressions in surgical wound healing

**DOI:** 10.1038/s41598-025-13924-1

**Published:** 2025-08-12

**Authors:** Ayman Mohammed El Makakey, Mohammed H. Hassan, Nehad A. Abo-zaid, Bakheet E. M. Elsadek, Mahmoud A. Hifny, Radwa Mahmoud Elsharaby, Mohammed E. Ali

**Affiliations:** 1 Department of Physical Therapy for Surgery, Faculty of Physical Therapy, Benha University, Benha, Egypt; 2 Department of Physical Therapy for Surgery, Faculty of Physical Therapy, Alsalam University, Kafr El Zayat, Egypt; 3 Department of Medical Biochemistry, Faculty of Medicine, South Valley University, Qena, 83523 Egypt; 4 Department of Physical Therapy for Pediatrics, Faculty of Physical Therapy, South Valley University, Qena, 83523 Egypt; 5 Department of Physical Therapy for Pediatrics, Faculty of Physical Therapy, Badr University in Assuit (BUA), Assiut, Egypt; 6 Department of Biochemistry and Molecular Biology, Faculty of Pharmacy, Assiut Branch, Al-Azhar University, Assiut, 71524 Egypt; 7 Department of Plastic Surgery, Faculty of Medicine, South Valley University, Qena, 83523 Egypt; 8 Department Clinical Pathology, Faculty of Medicine, Tanta University, Tanta, Egypt; 9 Department of Physical Therapy for Surgery , Faculty of Physical Therapy, South Valley University, Qena, 83523 Egypt; 10 Department of Medical Biochemistry, Faculty of Medicine, South Valley University, Qena, 83523 Egypt

**Keywords:** Microcurrent therapy, Low level laser therapy, Matrix metalloproteinases and the tissue inhibitors of metalloproteinases, Wound healing, Clinical trial, Comorbidities, Disability

## Abstract

**Purpose:**

The purpose of this study was to compare the modulation effects of Microcurrent Therapy (MT) and Low-Level Laser Therapy (LLLT) on Matrix Metalloproteinases (MMPs) and tissue inhibitors of Metalloproteinases (TIMPs) expressions during healing of surgical wounds using appendectomy wound as a model.

**Methods:**

Ninety patients who recently underwent appendectomy were randomly divided into 3 main groups of equal numbers. All cases in the three groups received ordinary medical therapy. Moreover, group A (MT group) received Microcurrent Therapy for 20 min. In addition to a designed physical therapy treatment protocol for 20 min. Group B (LLLT group) received Low-Level Laser Therapy for 20 min., plus the same designed physical therapy treatment protocol for 20 min. Group C (placebo group) received placebo shame LLLT for 20 min. plus the same designed physical therapy treatment protocol for 20 min. Enzyme-linked immunosorbent assay (ELISA) and Western Blot Technique (WBT) were used to determine expression levels of MMP-8, MMP-9, and TIMP-1 at the beginning of treatment and after the end of twelve successive sessions.

**Results:**

Following therapies, results showed a statistically significant decrease in the MMP-8 and MMP-9 expressions with significantly increased expression levels of TIMP-1 in each group separately (*P* < 0.05). These changes in the expression levels towards proper healing of surgical wounds were more obvious in MT and LLLT groups compared to the placebo group, with significantly better effect in the LLLT group compared to the MT group .

**Conclusion:**

Microcurrent therapy and low-level laser therapy have a notable impact in improving wound healing process as they can significantly affect the expression levels of matrix metalloproteinases and tissue inhibitors of metalloproteinases towards good prognosis of healing process and decreasing possible wound healing complication, with superior effect of low-level laser therapy.

**Supplementary Information:**

The online version contains supplementary material available at 10.1038/s41598-025-13924-1.

## Introduction

Appendicitis is the leading cause of emergency abdominal surgery in both adults and children, with over 180,000 people and 70,000 adolescents undergoing appendectomy each year in the United States. Coagulation, immune system abnormalities, diet, chronic diseases, drugs, and suturing procedures are some of the factors that affect normal surgical wound healing^1^.

Wound healing in its acute phase is considered as a complex active process forming a dynamic and coordinated sequence like a cascade which involve both cellular and molecular responses act in four phases that could be overlapped starting with hemostasis followed by inflammatory phase then granulation tissue formation ending with remodeling phase^2^. Numerous molecules including cytokines, growth factors, proteinases, and their inhibitors regulate these processes. Normal interactions of these molecules lead to progress of the acute wound through the phases of healing^3^.

Matrix Metalloproteinases (MMPs) are one of the most active molecules formed of zinc endopeptidases act in all stages of wound healing process removing all destructed proteins also the remnant of temporal ECM that could be active during the inflammatory phase and destruct the basement membrane of the capillaries allowing new angiogenesis and active cells to migrate in proliferation phase.it also affect the remodeling phase and its tissues by its contraction^4^as well as they control the activity of some growth factors involved in wound healing processes^5^. –^6^.

Matrix Metalloproteinases are categorized in subdivisions groups depending on their specificity. MMP-9 affects the wound closure when expressed on migrating keratinocytes at its leading edges^7^ and demonstrates a significant role in keratinocyte migration^8^as well as regulating angiogenesis^9^. On the other hand, MMP-8 (collagenase-2) plays an important role in cutaneous wound healing^10^ as it is the most abundant interstitial collagenase in human cutaneous excisional wounds^11^.

Although the essential roles of MMPs in normal skin wounds healing cannot be underestimated, elevated levels or uncontrolled activity of MMPs is associated with poor wound healing^12^. The excessive proteinases are responsible for destruction of the growth factors and extracellular matrix proteins, which resulted in dehiscence of acute wounds^13^. As a result, MMPs could disturb the wound healing if not found in adequate amounts^14^.

The main regulatory role of MMPs in wound healing are the specific inhibitory one which is called the tissue inhibitors of metalloproteinases (TIMPs) which have four different subtypes that inhibit and regulate the action of MMPs^15^. The imbalance between matrix proteins (MMPs) and their inhibitors TIMPs could lead to degradation of the ECM matrix with subsequent wound healing impairment^16^. The loss of balance between the ratio of MMPs and its inhibitory TIMP leads to degradation of the ECM matrix with subsequent wound healing impairment^16^.

Several physiotherapy modalities are being used to improve the quality of regenerative process^17^speed up the healing process, acting upon the interaction between external energetic stimuli and the biological tissue (biostimulation), promoting an increase in cellular activities during the healing process^18^ such as laser therapy, Low-Level Light Therapy, blue light emission diode, photodynamic therapy, electrical stimulation, ultrasound therapy, low frequency pulsed electromagnetic fields, and biophotonic therapy^19^.

The low-intensity laser therapy is among the modalities used, which can impact on all phases of wound healing by modulation of the inflammatory process, stimulation of collagen synthesis, and modulation of MMP activity^20^. Therefore, it can be used successfully in improving skin wound healing in humans and animals^21^. In addition, Micro current external application has a positive effect on wound healing^22^. It enhances wound healing process through enhancement of angiogenesis^23^re-epithelialization by facilitating migration of keratinocytes and controlling fibroblast activity^24^. Consequently, both MT and LLLT can be used for enhancing wound healing and also decrease the hazards of wound healing complications However, it has not been established which modality could be fastest and more effective^25,26^. So, our study aimed to investigate and compare the efficacy of LLLT and MT on metalloproteinases 8, 9, and TIMP-1 as an indicator and predictor factor of wound healing. We hypothesized that LLL and MT can positively influence the repair process of acute cutaneous wounds through modulation of the mediators of the healing process.

## Patients and methods

### Study design

A double-blinded randomized controlled trail was carried out in the Physical Therapy Faculty’s outpatient clinic, South Valley University during the period, from April 2023 to December 2024. The study received approval from the Faculty of Medicine’s clinical research ethics committee, South Valley University under the protocol No. (SVU-MED-PlS013-4-23-3-371) and additionally, it was listed on ClinicalTrials.gov (https://clinicaltrials.gov/study/NCT05326022) and had identifying number (NCT05326022), first posted date was (13\04\2022). Prior to beginning, the researchers explained the study’s protocol to each patient and got their informed consent to join the study procedures, to guarantee their contentment and to be advised that their data would be kept private and that they have the freedom to leave the study at any time without incurring any fees. The study followed the Declaration of Helsinki’s ethical guidelines for human research.

### Study population

The study was conducted on ninety patients with recently undergoing appendectomy. After they receive a clinical diagnosis, a surgeon referred them. They were of both genders and their ages were between thirty and seventeen years old, had stable medical and psychological status. We excluded from our study patients with an unsteady medical state, use of medications known to interfere with wound healing, especially those with cardiovascular problems, mental retardation, and uncooperative.

### Randomization

**Fig. 1:**
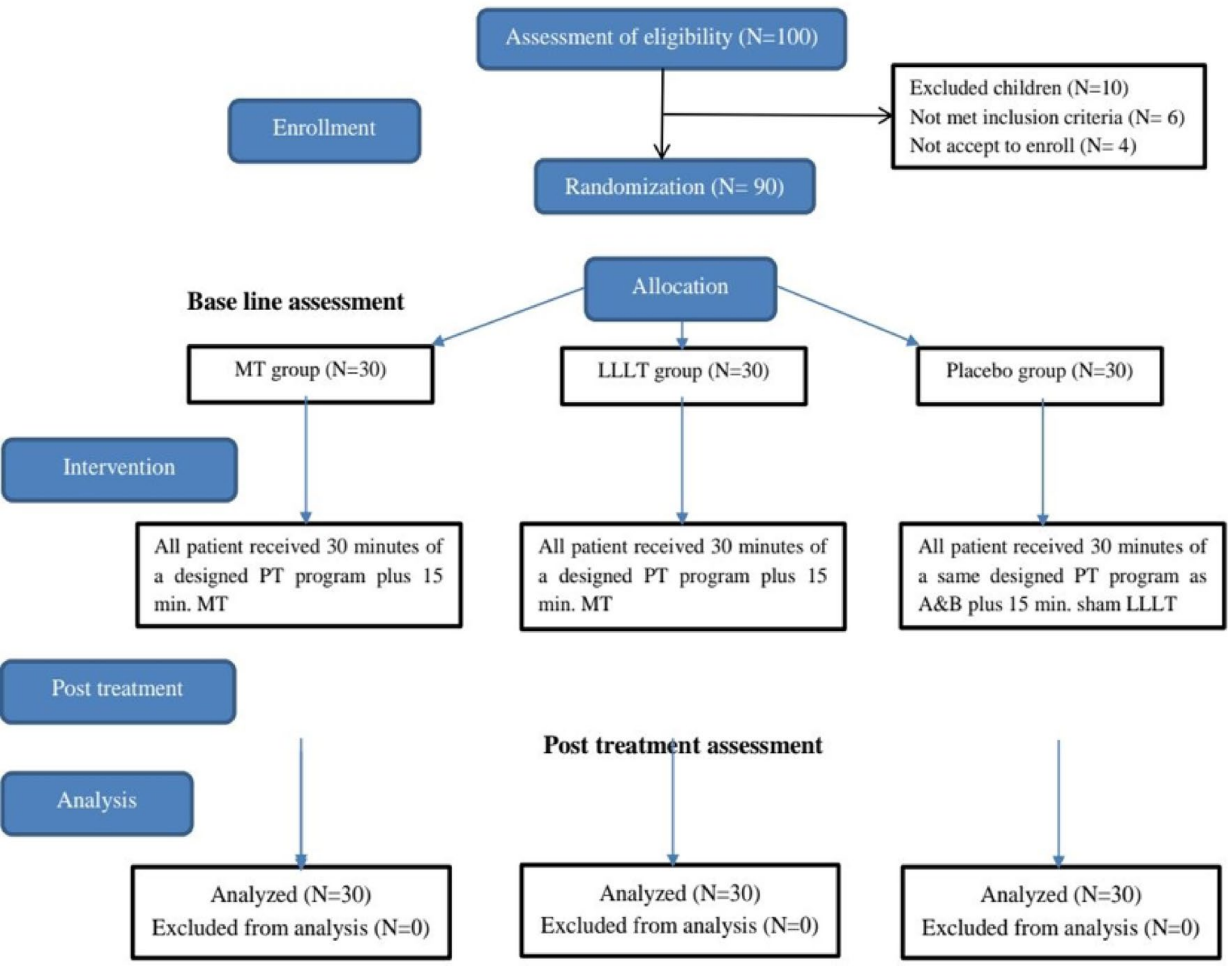
Flowchart of children randomization.

One hundred patients with recently undergoing appendectomy were first assessed for eligibility. Six patients failed to meet the requirements for inclusion, four declined to sign up. The remaining ninety patients were classified randomly into three groups with equal numbers by a blind independent researcher. To guarantee the distribution of equal numbers in each group, randomization was restricted to permuted blocks of varying sizes. Until it was needed, each randomly permuted block was kept in a locked drawer as opaque, sealed envelopes with sequential numbers. The researcher opened the next envelope in order that each participant had been formally included in the trial and in the patient’s presence. Everyone involved was blinded to the study’s hypothesis. The randomization protocol was kept secure by the research assistant. The patients and investigators responsible for follow-up were blinded to the randomized allocations until the completion of final statistical analyses. After randomization, no subjects dropped out of the research. The randomization diagram of patients is shown in **(**Fig. 1).

### Intervention

All cases in different groups received the conventional anti-scar measurement including application of silicon creams starting immediately after stitches removal^27^. Moreover, group **A** (MT group) received Microcurrent Therapy for 15 min. **)PhysioGoPHG100A-03/N1/AY)** with frequency 0.3 to 50 Hz and intensity 300 to 600 ^28^ in addition to a designed physical therapy treatment protocol for 20 min. including stretching exercises for quadriceps, hamstrings, gastrocnemius, and upper Trap. Other suggested exercises include sit to stand, ankle pumps, glute squeezes, seated knee extension, heel raises, shoulder blade squeezes, and pelvic floor contraction with breath^29^. The patients were instructed to perform graduated abdominal strengthening exercises in the form of activation of rectus abdominis, posterior pelvic tilt, rotational planks, and abdominal crunch on Swiss ball with elastic resistance^30^. Group **B** (LLLT group) received Low Level Laser Therapy for 20 min. **(SKW2-450&400**,** SK2-450&400)**, HeNe (632.8 nm wavelength, dose of 5 J/cm^2^ and power 10.53 mW/cm^2^), utilized three times / week^31^ in addition to the same designed physical therapy treatment protocol for 20 min. Group **C** (placebo group) received placebo shame LLLT for 20 min. (For sham LLLT; the same procedures were done as in group B but the stimulator was turned off) plus the same designed physical therapy treatment protocol for 20 min. All cases underwent the treatment regimen 3 times / week for three successive months^30^^31^. carried out by a particular therapist, but another therapist performed the assessment to prevent bias.

All patients in all groups were assessed for the expression levels of MMP-8, MMP-9, and TIMP-1 at base line and after twelve successive sessions of the applied treatment protocol.

### Outcome measures

First, 5 milliliters of blood were drawn and centrifuged at 3000 rpm for 5 min. Western Blot Technique (WBT) and Enzyme-linked immunosorbent assay (ELISA) were used to separate and store serum samples at -20 ◦C in order to determine the protein content of MMP-8, MMP-9, and TIMP-1^32^.

### Western blot technique (WBT)

MMP-8, MMP-9, and TIMP-1 protein expressions were assessed by Western Blotting. Sodium dodecyl sulfate (SDS)–polyacrylamide gel electrophoresis (PAGE) was used to separate proteins (20 µg from each sample). The separated proteins were then transferred to PVDF membranes using Trans-Blot Turbo (Bio-Rad, CA, USA) for seven minutes at 25 V. After that, 3% (w/v) bovine serum albumin (BSA) in 20 mM Tris Buffered Saline with 0.1% (v/v) Tween (TBST) was used to block the membrane. The membranes were treated with anti-β-actin (1:1000, Abcam, Cambridge, UK, ab8226) as the primary antibody for an entire night at 4 °C. After that, secondary antibodies conjugated with horseradish peroxidase (HRP) (1:1000; Sigma-Aldrich Chemicals, St. Louis, MO, USA) were incubated for two hours at room temperature. The blot was treated with the chemiluminescent substrate (Bio-Rad, CA, USA) in accordance with the manufacturer’s instructions. Following incubation with HRP-conjugated secondary antibody and the chemiluminescent substrate (Bio-Rad, CA, USA), images of the bands were then seen using a CCD camera-based imager (Supplementary Figs. 1–3). Following the normalization of β-actin protein expression (as the housekeeping protein), the data were finally evaluated^32^. The analysis was repeated (number of assessed patients in each group = 3) to assure reproducibility of results. Quantification was performed using image J software, and expressed as the band density relative to that of β-actin.

### Enzyme-linked immunosorbent assay (ELISA)

Serum levels of MMP-8, MMP-9, and TIMP-1 were measured using the sandwich enzyme-linked immune-sorbent assay methodology of the Enzyme-linked Immunosorbent Assay Kit for MMP-8, MMP-9, and TIMP-1 from Chongqing Biospes Co. with cat no. (BEK1161, BEK1162, and BEK1208), respectively^32^.

At baseline and after twelve consecutive sessions of the implemented treatment plan, all patients in all groups were evaluated using the Western Blot Technique (WBT) and the Enzyme-linked Immunosorbent Assay (ELISA).

### Sample size

A preliminary power analysis was applied using power program 3.1.9 (version 3.1, software) to calculate the sample size for this study depending on F tests (MANOVA: Special effects and interactions), Type I error (α) = 0.05, power (1-α error probability) = 0.80, Pillai V = 0.13 and effect size f2 (V) = 0.0695 with 3 independent groups comparison for MMP-8, MMP-9 and TIMMP outcome measures. A minimum sample size of twenty-six patients in each group was appropriate.

### Statistical analysis

For each variable, the Shapiro-Wilk test was used to verify that the data had a normal distribution. To determine if groups were homogeneous, Levene’s test for homogeneity of variances was used. To compare the effects of each group on MMP-8, MMP-9, and TIMP-1, a mixed MANOVA was used. The Bonferroni correction was used in post-hoc tests for the multiple comparisons that followed. All statistical tests had a significance level of *p* < 0.05. IBM SPSS, Chicago, IL, USA’s statistical program for social studies (SPSS) version 25 for Windows was used for all statistical analyses.

## Results

Demographic data for all patients is demonstrated in (Table 1). Between the control and WBV groups, no statistically significant variations were seen in sex, age, weight, height, as well as BMI (*p* > 0.05).

**Table 1:** Patients demographic data among both groups.

Variables	Group A	Group B	Group C	T test	*P* value
Sex (No. %)					
Male n (%)	17 (56.7%)	19 (63.3%)	18 (60%)	0.283	0.870
Female n (%)	13 (43.3%)	11 (36.7%)	12 (40%)		
Age (year, Mean ± SD)	15.32 ± 0.84	15.62 ± 1.03	15.48 ± 0.94	0.750	0.475
Height (cm, Mean ± SD)	160.40 ± 6.51	159.00 ± 7.05	160.28 ± 6.85	0.387	0.680
Weight (kg, Mean ± SD)	56.27 ± 2.74	56.34 ± 2.77	55.94 ± 2.67	0.189	0.828
BMI (kg/m^2^, Mean ± SD)	21.96 ± 1.87	22.39 ± 2.08	21.88 ± 2.10	0.561	0.573

### Effect of treatment on MMP-8, MMP-9 and TIMP-1

The results of a mixed MANOVA showed that treatment and time significantly interacted (F = 58.19, *p* = 0.001, η^2^ = 0.67). There was a significant main effect of time (F = 2566.57, *p* = 0.001, η^2^ = 0.98). There was a significant main effect of treatment (F = 48.17, *p* = 0.001, η^2^ = 0.63).

**Fig. 2:**
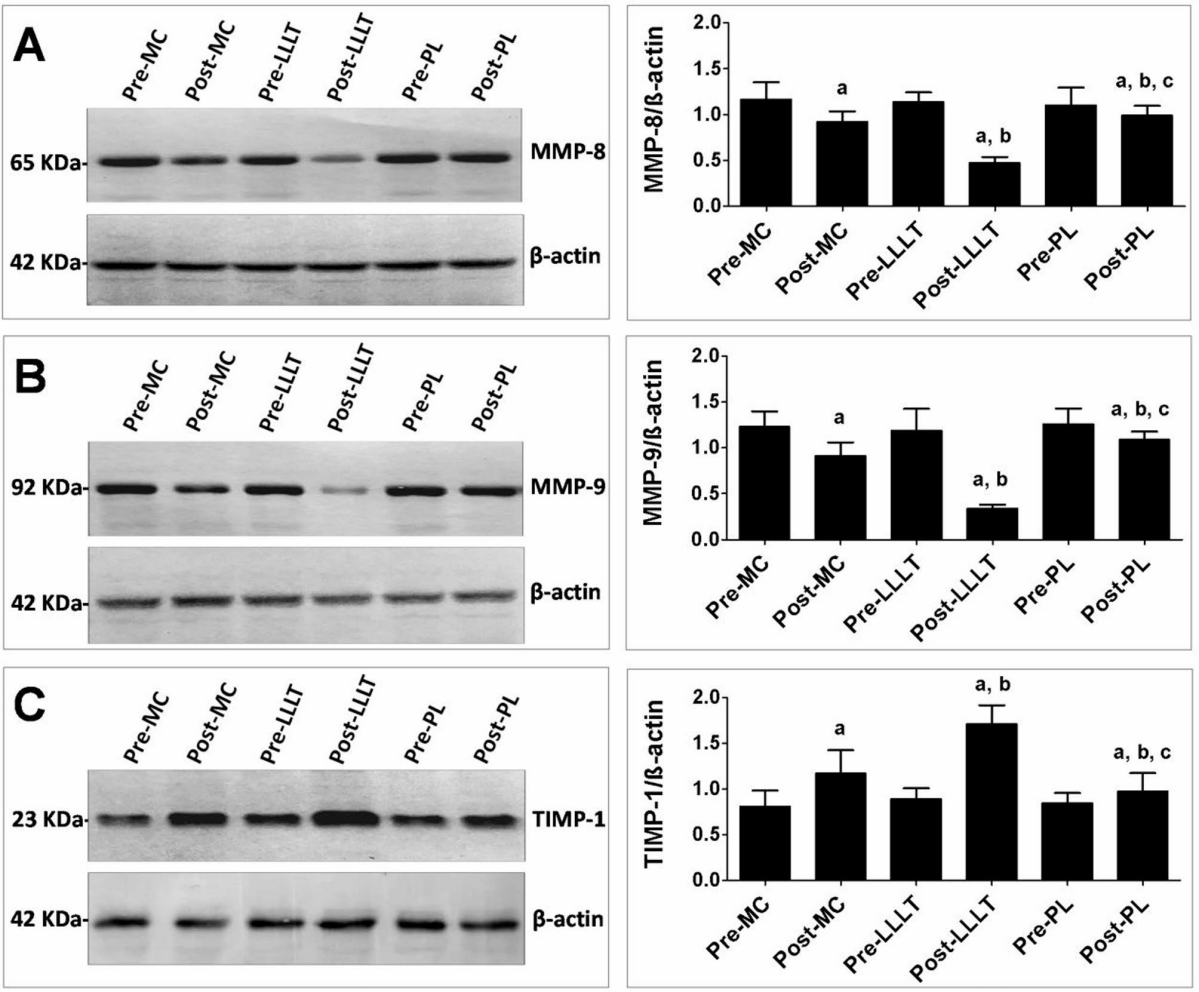
Representative Western blot analysis of MMP-8 (**A**), MMP-9 (**B**), and TIMP-1 (**C**) expressions in skin tissue homogenates of different groups. Data are presented as mean ± SD (*n* = 3). Differences were considered statistically significant at *p* < 0.05. β-actin was used in parallel as internal control. The right panels represent corresponding quantification of each gel analysis measured by image J software and expressed as β-actin ratio a indicates significant change from each corresponding pre-treatment group; b indicates significant change from the Post-MC group; c indicates significant change from the Post-LLLT group.

After treatment, the three groups’ MMP-8 and MMP-9 levels significantly decreased, but their TIMP-1 levels significantly increased as compared to their pretreatment levels in each group (*p* < 0.01 for all) (Table 2; Fig. 2).

In comparison to group C, MMP-8 and MMP-9 significantly decreased in groups A and B (*p* < 0.001). There was a notable increase TIMP-1 in group A and B when compared with that of group C, (*p* < 0.001) using ELISA assays and Western blot analysis, (Table 3; Fig. 2).

**Table 2:** Mean MMP-8, MMP-9 and TIMMP before and after treatment of three different groups.

Variables	Group A	Group B	Group C
	Mean ± SD	Mean ± SD	Mean ± SD
MMP-8			
Pre-treatment	0.52 ± 0.02	0.53 ± 0.02	0.53 ± 0.03
Post treatment	0.35 ± 0.03	0.36 ± 0.04	0.44 ± 0.03
MD	0.17	0.17	0.09
95% CI	0.15: 0.18	0.15: 0.18	0.08: 0.10
*P* value	*p* = 0.001	*p* = 0.001	*p* = 0.001
MMP-9			
Pre-treatment	1.09 ± 0.06	1.11 ± 0.06	1.10 ± 0.07
Post treatment	0.74 ± 0.03	0.73 ± 0.04	0.92 ± 0.03
MD	0.35	0.38	0.18
95% CI	0.32: 0.38	0.36: 0.41	0.15: 0.21
*P* value	*p* = 0.001	*p* = 0.001	*p* = 0.001
TIMP-1			
Pre-treatment	0.93 ± 0.04	0.95 ± 0.03	0.94 ± 0.05
Post treatment	2.16 ± 0.14	2.08 ± 0.15	1.52 ± 0.11
MD	−1.23	−1.13	−0.58
95% CI	−1.27: −1.18	−1.18: −1.09	−0.62: −0.52
*P* value	*p* = 0.001	*p* = 0.001	*p* = 0.001

SD, Standard deviation; MD, Mean difference; CI, Confidence interval; p value, Probability value.

There was no significant difference in MMP-8, MMP-9 and TIMP-1 between group A and B (*p* > 0.05) using ELISA assays (Table 3), but Western blot analysis showed more significant effect of LLLT compared to MT on the expression levels of MMP-8, MMP-9 and TIMP-1 (*p* < 0.05) (Fig. 2).

**Table 3:** Comparison of MMP-8, MMP-9 and TIMP-1 among three different groups (A, B, C) after treatment.

Outcome	Group A vs. B	*p* value	Group A vs. C	*p* value	Group B vs. C	*p* value	η2
	MD (95% CI)		MD (95% CI)		MD (95% CI)		
MMP-8	−0.01 (−0.03: 0.01)	0.22	−0.09 (−0.11: −0.07)	0.001	−0.08 (−0.10: −0.06)	0.001	0.63
MMP-9	0.01 (−0.01: 0.03)	0.34	−0.18 (−0.20: 0.16)	0.001	−0.19 (−0.21: −0.18)	0.001	0.87
TIMP-1	0.08 (−0.01: 0.16)	0.08	0.64 (0.56: 0.73)	0.001	0.56 (0.48: 0.65)	0.001	0.82

MD, Mean difference; CI, Confidence interval; p value, Probability value; η^2^Partial Eta Squared.

## Discussion

Wound healing process warrants controlling the degradation of ECM so any disturbance of its balance between its formation and degradation that could affect normal wound healing processes and its efficacy therefore the presence of subsequent complications of wound healing^33^. One of the main proteases that involved in this regulation is MMPs. There are many pathological conditions affect the process of organization of extracellular matrix like chronic wound shown disturbance in formation of MMPs or/and change the balance of the enzymes/inhibitors ratio^34^.

The MMPs long overexpression degrades essential proteins and delays the healing process^35^. However, excessive levels of proteases are not the only mechanism that impairs wound healing. One of the most essential factors in the process of wound healing and repair is the balance between the ratio of MMPs and TIMPs as the last affect the modulation of MMPs^36^.

Bellayr et al., and Haubner et al., found that overexpression of MMPs along with decreased levels of TIMPs can affect repair of the skin wound^37,38^. Moreover, Gill and Parks reported the importance in the balance of expression between MMPs and TIMPs in wound healing^39^. The high levels of MMP-1, MMP-8, and MMP-9 expressions in cases of venous wounds with absence of TIMPs were reported^6^.

Muller et al. found the higher level of activated MMP-9 is harmful while the high level of TIMP-1 was reported in group of good healing results^40^. Interestingly Ladwiget et al., study demonstrated that the positive ratio of MMPs/TIMPs gave poor healing results in patient with pressure ulcers^41^.

Lobmannet et al., reported a significant decline in the ratio between MMP-9/TIMP-2 in patients with diabetes who displaying an accelerating wound healing when managed with a protease modulating matrix^42^. Ultimately, there is a generally accepted idea that any modality that decreases the ratio of MMPs over TIMPs can protect the ECM and minimize proteolysis.

The PBMT has been shown to affect the cutaneous wounds healing by changing of this process mediators, like vascular endothelial growth factor (VEGF), collagen^43^pro-inflammatory cytokines^44^ and production of MMPs, TIMP. There are several clinical studies that have shown an improvement in the wound-healing process with the LLLT using^21^ and MC^22^. However, this previous research did not assess the effects of these therapies on proteinase synthesis and/or activities in acute wound healing.

The reduction of proteinase activities has provided strong evidence to direct wounds toward a healing trajectory^35^ and as a result, the evaluation of the levels of MMPs and TIMPs and MMP/TIMP ratio are important to compare two different energy densities (MT, LLLT application).

In the current study, the effect of LLLT and microcurrent therapy on MMPs and TIMP-1 levels were investigated in post-surgical wounds. MMPs (including MMP-8 and MMP-9 activity), and TIMP-1 expression levels were measured, which reflect the inflammatory proteases activity in the wound. Their assays were done by using of both Enzyme-linked immunosorbent assay (ELISA) test and Western Blot Technique (WBT) technique as WBT with its high levels of specificity, sensitivity and versatile application so it can detect protein in complex and different biological samples while ELISA gives results that seems to be clear and could interpret easily so WBT was used to confirm the results generated from ELISA and so provide a reliable indication of the proteolytic status of the wound. Our results of all groups group A (MC group) that received Microcurrent Therapy in addition to both medical and a designed physical therapy treatment protocol as well as group B (LLLT group) that received Low Level Laser Therapy and the same physical therapy and medical treatment of group A and group C (placebo group) received placebo shame LLLT and the same designed physical and medical therapy) showed no statistically significant differences between them in pre application measurements values. A statistical significant increase in TIMP-1 levels and a statistical significant reduction in the level of proteases MMPs, (MMP-8, and MMP-9) in post-treatments measurements values of all groups compered to pre-treatments measurements ones were observed. Statistically significant differences were found in post- treatment values and measurements of both group A and group B compared to the results of group C which were higher in group A and group B than group C while there were no statistically significant differences in between post-treatment measurements values of both group A and group B using ELISA assays with significant higher effect of LLLT than MT using WBT. The observation of TIMP-1 expressions increased while MMP-8 and MMP-9 value decreased post application of MT group and LLLT and placebo group compared to its pre application proves that the TIMP modulates MMP expressions and promotes wound healing and denoting decreased wound healing complications. This also shows that both LLLT and MC had a potent effect in biomodulation of the TIMP-1 and MMPs expression as compared to control group C, with superior effect of LLLT.

The outcomes of this study are concurrent with those of Da Silva et al.^45^ which had shown decreases in expressions of MMP-2 and MMP-9 in healing of wounds in diabetic animals undergo a single application of LLLT. Furthermore, Alves et al., found that the LLLT reduced metalloproteinase 9 and is efficient in the tissue repair^46^. Ayuk et al. study showed that relatively low TIMP levels and the elevated levels of MMP-1, MMP-8, and MMP-9 were observed with poor wound healing^47^. Oton-Leite et al. demonstrated that the reduced levels MMP-9/TIMP-2 after treatments of patients with low level laser therapy as compared to the untreated group^48^. Kim et al. study showed the modulating effects of LLLT on gene expression of MMPs (MMP-1, MMP-2, MMP-8, MMP-9, and MMP-13) in the rat’s periodontal ligament concurrent with inhibiting the immunoreactivity of TIMP-1^50^. Barbara Kapeller et al. also noted the effectiveness of microcurrent on reverse remodeling in cardiomyocytes in both vitro and vivo with elevated levels of on MMP-2, MMP‐9, and decreased levels of TIMP‐3, and TIMP‐4 mRNA and the expression of their proteins also were observed^50^.

The effect of LLLT to promote wound healing is demonstrated with several physiological properties due to its application as its stimulatory effect on Mitochondrial Respiration and ATP, Nitric Oxide inhibition and enhancing Reactive Oxygen Species (ROS) and Gene Transcription^51,52^ also microcurrent use promote wound healing through increasing the rates of tissue synthesis, neural and promoting angiogenesis and increase adenosine triphosphate (ATP) production which stimulates mitochondrial biogenesis^53^.

But with demonstrating their effect on matrix metalloproteinases and the tissue inhibitors of metalloproteinases ratio we can conclude that the using of LLLT and MT not only stimulate and promote wound healing but also help in decreasing complications of wound healing as poor wound healing and delayed wound healing and scar formation so its use as a routine treatment in caring of wound in vulnerable cases as elder patients, patients with chronic morbidity and burned patients could be helpful in decrease expected wound healing complications.

The relatively small sample size, single center study, and lack of long term follow up of the included patients were the main limitations of the study.

## Conclusion

Low-level laser therapy has a significant effect which appears to be more potent compared to Microcurrent therapy in improving the wound healing process as they can lower MMPs with significant upregulation of TIMP-1 which indicates good prognosis of the healing process and decrease possible wound healing complications. So possible use of Microcurrent therapy or preferably Low-level laser therapy as add-on therapy may be promising and helpful in the healing of surgical wounds, but this needs more experimental research to confirm the findings of the current study.

## Supplementary Information

Below is the link to the electronic supplementary material.

Supplementary Material 1

## Data Availability

The datasets used and/or analyzed during the current study are available from the corresponding author upon reasonable request, after obtaining the permission of our institute.

## References

[1] Masraini Daulay, N. & Angraini Simamora, F. Efektivitas mobilisasi Dini Terhadap Penyembuhan Luka Paska Operasi Apendiktomi. *J. Educ. Dev.* **7** (4), 245 (2019).

[2] Dickinson, L. E. & Gerecht, S. Engineered biopolymeric scaffolds for chronic wound healing. *Front. Physiol.* **7**, 341 (2016). 27547189 10.3389/fphys.2016.00341 PMC4975021

[3] Trengove, N. J. et al. Analysis of the acute and chronic wound environments: the role of proteases and their inhibitors. *Wound Repair. Regen*. **7** (6), 442–452 (1999). 10633003 10.1046/j.1524-475x.1999.00442.x

[4] Vitlianova, K., Georgieva, J., Milanova, M. & Tzonev, S. Blood pressure control predicts plasma matrix metalloproteinase-9 in diabetes mellitus type II. *Arch. Med. Sci.* **11** (1), 85–91 (2015). 25861293 10.5114/aoms.2015.49208 PMC4379372

[5] Sabino, F. & Keller, U. auf dem Matrix metalloproteinases in impaired wound healing. Metalloproteinases In Medicine. ; 2:1–8 (2015).

[6] Amato, B. et al. Role of matrix metalloproteinases in non-healing venous ulcers. *Int. Wound J.* **12** (6), 641–645 (2015). 24164799 10.1111/iwj.12181 PMC7950624

[7] Castaneda, F. E. et al. Targeted deletion of metalloproteinase 9 attenuates experimental colitis in mice: central role of epithelial-derived MMP. *Gastroenterology* **129** (6), 1991–2008 (2005). 16344067 10.1053/j.gastro.2005.09.017

[8] Hattori, N. et al. MMP-13 plays a role in keratinocyte migration, angiogenesis, and contraction in mouse skin wound healing. *Am. J. Pathol.* **175** (2), 533–546 (2009). 19590036 10.2353/ajpath.2009.081080 PMC2716954

[9] Heljasvaara, R. et al. Generation of biologically active endostatin fragments from human collagen XVIII by distinct matrix metalloproteases. *Exp. Cell. Res.* **307** (2), 292–304 (2005). 15950618 10.1016/j.yexcr.2005.03.021

[10] Gutiérrez-Fernández, A. et al. Increased inflammation delays wound healing in mice deficient in collagenase-2 (MMP-8). *FASEB J.* **21** (10), 2580–2591 (2007). 17392479 10.1096/fj.06-7860com PMC2575772

[11] Hasty, K. A., Hibbs, M. S., Kang, A. H. & Mainardi, C. L. Secreted forms of human neutrophil collagenase. *J. Biol. Chem.* **261** (12), 5645–5650 (1986). 3007518

[12] McCarty, S. M. & Percival, S. L. Proteases and delayed wound healing. *Adv. Wound Care (New Rochelle)*. **2** (8), 438–447 (2013). 24688830 10.1089/wound.2012.0370 PMC3842891

[13] Cowin, A. J. et al. Effect of healing on the expression of transforming growth factor beta(s) and their receptors in chronic venous leg ulcers. *J. Invest. Dermatol.* **117** (5), 1282–1289 (2001). 11710945 10.1046/j.0022-202x.2001.01501.x

[14] Martins, V. L., Caley, M. & O’Toole, E. A. Matrix metalloproteinases and epidermal wound repair. *Cell Tissue Res.* **351** (2), 255–268 (2013). 22526628 10.1007/s00441-012-1410-z

[15] Visse, R. & Nagase, H. Matrix metalloproteinases and tissue inhibitors of metalloproteinases: structure, function, and biochemistry. *Circul. Res.* **92** (8), 827–839 (2003). 10.1161/01.RES.0000070112.80711.3D 12730128

[16] Overall, C. M. & L´opez-Ot´ın, C. Strategies for MMP Inhibition in cancer: innovations for the post-trial era. *Nat. Rev. Cancer*. **2** (9), 657–672 (2002). 12209155 10.1038/nrc884

[17] Okuni, I. Phototherapy in rehabilitation medicine. *Masui* **61** (7), 700–705 (2012). 22860298

[18] Santos, V. N. S., Ferreira, L. M., Horibe, E. K. & Duarte, I. S. Electric microcurrent in the restoration of the skin undergone a trichloroacetic acid peeling in rats. *Acta Cir. Bras.* **19** (5), 466–470 (2004).

[19] Fernández-Guarino, M. et al. The role of physical therapies in wound healing and assisted scarring. *Int. J. Mol. Sci.* **24** (8), 7487 (2023). 37108650 10.3390/ijms24087487 PMC10144139

[20] Leal-Junior, A. D. S. A. & Alves, E. C. Wound-healing effects of low-level laser therapy in diabetic rats involve the modulation of MMP-2 and MMP-9 and the redistribution of collagen types I and III. *J. Cosmet. Laser Ther.* **15** (4), 210–216 (2013). 23463906 10.3109/14764172.2012.761345

[21] Zanotti, G. B., Oliveira, P. I., Reis, S. F. S., Silva, F. S. & Araújo, A. R. Efeitos do laser de baixapotênciasobre a Regeneração Da cartilagemnaosteoartrose. *Rev. Fisio Bras.* **12** (2), 139–146 (2011).

[22] Luanraksa, S. et al. An MMP/TIMP ratio scoring system as a potential predictive marker of diabetic foot ulcer healing. *J. Wound Care 2018 2* ;**27**(12):849–855 . 10.12968/jowc.2018.27.12.849 30557113

[23] Park, R. et al. The effect of microcurrent electrical stimulation on the foot blood circulation and pain of diabetic neuropathy. *J. Phys. Therapy Sci.* **23**, 515–518 (2011).

[24] Banerjee, J. et al. Improvement of human keratinocyte migration by a redox active bioelectric dressing. *PLoS One*. **9** (3), e89239 (2014). 24595050 10.1371/journal.pone.0089239 PMC3940438

[25] Reddy, G. K., Stehno-Bittel, L. & Enwemeka, C. S. Laser photostimulation accelerates wound healing in diabetic rats. *Wound Repair. Regen*. **9** (3), 248–255 (2001). 11472621 10.1046/j.1524-475x.2001.00248.x

[26] Woodruff, L. D. et al. The efficacy of laser therapy in wound repair: a meta-analysis of the literature. *Photomed. Laser Surg.* **22** (3), 241–247 (2004). 15315732 10.1089/1549541041438623

[27] Ogawa, R. The most current algorithms for the treatment and prevention of hypertrophic scars and keloids: A 2020 update of the algorithms published 10 years ago. *Plast. Reconstr. Surg.* **149** (1), 79e–94e (2022). 34813576 10.1097/PRS.0000000000008667 PMC8687618

[28] AHMED, M. et al. Effect of microcurrent electrical stimulation on microcirculation of chronic leg ulcers. *Med. J. Cairo Univ.* **91** (3), 273–281 (2023).

[29] Mohammed, E., Ali, Haidy, N., Asham, N. & Abo-Halawa, Nesma, M. Allam – Russian stimulation in addition to graduated abdominal exercises versus graduated abdominal exercises only on muscle strength after ventral hernioplasty: A randomized controlled trial. *Fizjoterapia Polska*. **21** (4), 72–77 (2021).

[30] Salah, A. M., Mohamed, G. A., Mohamed, M. & Walid, A. Effect of preoperative abdominal training on abdominal muscles strength outcomes after ventral hernia repair. *Med. J. Cairo Univ.* **86** (8), 4495501 (2018).

[31] Landau, Z. & Schattner, A. Topical hyperbaric oxygen and low energy laser therapy for chronic diabetic foot ulcers resistant to conventional treatment. *Yale J. Biol. Med.* **74** (2), 95–100 (2001). 11393266 PMC2588691

[32] El-Sisi, A. E. E., Sokar, S. S., Shebl, A. M., Mohamed, D. Z. & Abu-Risha, S. E. Octreotide and melatonin alleviate inflammasome-induced pyroptosis through Inhibition of TLR4-NF-κB-NLRP3 pathway in hepatic ischemia/reperfusion injury. *Toxicol. Appl. Pharmacol.* **410**, 115340 (2021). 33264646 10.1016/j.taap.2020.115340

[33] Kandhwal, M. et al. Role of matrix metalloproteinase in wound healing. *Am. J. Transl Res.* **14** (7), 4391–4405 (2022). 35958464 PMC9360851

[34] Nalbone, G., Alessi, M. C. & Juhan-Vague, I. Systeme fibrinolytique, metalloprotease set pathologie vasculaire. *Medecine/Sciences* **17**, 170–176 (2001).

[35] Gibson, D., Cullen, B., Legerstee, R., Harding, K. G. & Schultz, G. MMP’s made easy. *Wounds Int.* ; 1. (2009).

[36] McCarty, S. M. & Percival, S. L. Proteases and delayed wound healing. *Adv. Wound Care (New Rochelle)*. **2**, 438–447 (2013). 24688830 10.1089/wound.2012.0370 PMC3842891

[37] Bellayr, I. H., Mu, X. & Li, Y. Biochemical insights into the role of matrix metalloproteinases in regeneration: challenges and recent developments. *Future Med. Chem.* **1** (6), 1095–1111 (2009). 20161478 10.4155/fmc.09.83 PMC2794138

[38] Haubner, F. et al. A Co-Culture model of fibroblasts and adipose Tissue-Derived stem cells reveals new insights into impaired wound healing after radiotherapy. *Int. J. Mol. Sci.* **16** (11), 25947–25958 (2015). 26528967 10.3390/ijms161125935 PMC4661794

[39] Gill, S. E. & Parks, W. C. Metalloproteinases and their inhibitors: regulators of wound healing. *Int. J. Biochem. Cell. Biol.* **40** (6–7), 1334–1347 (2008). 18083622 10.1016/j.biocel.2007.10.024 PMC2746915

[40] Muller, M. et al. Matrix metalloproteinases and diabetic foot ulcers: the ratio of MMP-1 to TIMP-1 is a predictor of wound healing. *Diabet. Med.* **25** (4), 419–426 (2008). 18387077 10.1111/j.1464-5491.2008.02414.x PMC2326726

[41] Ladwig, G. P. et al. Ratios of activated matrix metalloproteinase-9 to tissue inhibitor of matrix metalloproteinase-1 in wound fluids are inversely correlated with healing of pressure ulcers. *Wound Rep. Reg.* **10**, 26–37 (2002). 10.1046/j.1524-475x.2002.10903.x 11983004

[42] Lobmann, R., Zemlin, C., Motzkau, M., Reschke, K. & Lehnert, H. Expression of matrix metalloproteinases and growth factors in diabetic foot wounds treated with a protease absorbent dressing. *J. Diabetes Complications*. **20**, 329–335 (2006). 16949521 10.1016/j.jdiacomp.2005.08.007

[43] Leal-Junior, A. D. S. A., Alves, E. C., Rambo, A. C., Dos Santos, C. S. & Vieira, S. A. De CarvalhoPde T Wound-healing effects of low-level laser therapy in diabetic rats involve the modulation of MMP-2 and MMP-9 and the redistribution of collagen types I and III. *J. Cosmet. Laser Ther.* **15** (4), 210–216 (2013). 23463906 10.3109/14764172.2012.761345

[44] Fukuda, T. Y., Tanji, M. M., Silva, S. R., Sato, M. N. & Plapler, H. Infrared low-level diode laser on inflammatory process modulation in mice: pro- and anti-inflammatory cytokines. *Lasers Med. Sci.* **28** (5), 1305–1313 (2013). 23179306 10.1007/s10103-012-1231-z

[45] Leal-Junior, A. D. S. A., Alves, E. C., Rambo, A. C., Dos Santos, C. S. & Vieira, S. A. De Carvalho PT Wound-healing effects of low-level laser therapy in diabetic rats involve the modulation ofMMP-2 andMMP-9 and the redistribution of collagen types I and III. *J. Cosmet. Laser Ther.* **15** (4), 210–216 (2013). 23463906 10.3109/14764172.2012.761345

[46] Alves, A. C. et al. Effect of low-level laser therapy on metalloproteinase MMP-2 and MMP-9 production and percentage of collagen types I and III in a Papain cartilage injury model. *Lasers Med. Sci.* **29** (3), 911–919 (2014). 23990219 10.1007/s10103-013-1427-x

[47] Ayuk, S. M., Abrahamse, H. & Houreld, N. N. The role of matrix metalloproteinases in diabetic wound healing in relation to photobiomodulation. *J. Diabetes Res.* ; 2897656. (2016). 10.1155/2016/2897656 PMC4893587 27314046

[48] Oton-Leite, A. F. et al. Effect of low-level laser therapy on chemoradiotherapy-induced oral mucositis and salivary inflammatory mediators in head and neck cancer patients. *Lasers Surg. Med.* **47** (4), 296–305 (2015). 25824475 10.1002/lsm.22349

[49] Kim, S. J., Kang, Y. G., Park, J. H., Kim, E. C. & Park, Y. G. Effects of low-intensity laser therapy on periodontal tissue remodeling during relapse and retention of orthodontically moved teeth. *Lasers Med. Sci.* **28** (1), 325–333 (2013). 22814894 10.1007/s10103-012-1146-8

[50] Kapeller, B., Mueller, J., Losert, U., Podesser, B. K. & Macfelda, K. Microcurrent stimulation promotes reverse remodelling in cardiomyocytes. *ESC Heart Fail.* **3** (2), 122–130 (2016). 27774272 10.1002/ehf2.12080 PMC5064659

[51] Farivar, S., Malekshahabi, T. & Shiari, R. Biological effects of low-level laser therapy. *J. Lasers Med. Sci.* **5** (2), 58–62 (2014). 25653800 PMC4291815

[52] Elshazly, M. & Askary, Z. M. Combined effect of negative pressure wound therapy and cryo-air therapy on diabetic wound healing. *SVU-International J. Med. Sci.* **5** (1), 58–67 (2022).

[53] Kolimechkov, S. et al. Physiological effects of microcurrent and its application for maximising acute responses and chronic adaptations to exercise. *Eur. J. Appl. Physiol.* **123** (3), 451–465 (2023). 36399190 10.1007/s00421-022-05097-w PMC9941239

