# Supplementary material for: Comparison of microcurrent and low level laser therapy on matrix metalloproteinases and tissue inhibitors of metalloproteinases expressions in surgical wound healing
Source: Sci Rep. 2025 Aug 12;15:29600. doi: 10.1038/s41598-025-13924-1 (PMC12343887; doi:10.1038/s41598-025-13924-1)
Supplement: Supplementary file 1 — Supplementary Material 1 [file 41598_2025_13924_MOESM1_ESM.docx]

**Supplementary materials**

**Comparison of Microcurrent and Low Level Laser Therapy on Matrix Metalloproteinases and tissue inhibitors of Metalloproteinases expressions in Surgical Wound Healing**

**Ayman Mohammed El Makakey^1,2^, Mohammed H. Hassan^3*^, Nehad A. Abo-zaid ^4,5^, Bakheet E.M. Elsadek^6^, Mahmoud A. Hifny^7^, Radwa Mahmoud Elsharaby^8^ ,** [**Mohammed E Ali**](https://pubmed.ncbi.nlm.nih.gov/?term=Ali+ME&cauthor_id=39441113)**^9^**

^1^Department of Physical Therapy for Surgery, Faculty of Physical Therapy, Benha University, Egypt.

^2^Department of Physical Therapy for Surgery, Faculty of Physical Therapy, Alsalam University, Egypt.

^3^Department of Medical Biochemistry, Faculty of Medicine, South Valley University, Qena 83523, Egypt.

^4^Department of Physical Therapy for Pediatrics, Faculty of Physical Therapy, South Valley University, Qena 83523, Egypt.

5Department of Physical Therapy for Pediatrics, Faculty of Physical Therapy, Badr University in Assuit, Assuit (BUA), Egypt.

^6^Department of Biochemistry and Molecular Biology, Faculty of Pharmacy, Al-Azhar University, Assiut Branch, 71524 Assiut, Egypt.

^7^Department of Plastic Surgery, Faculty of Medicine, South Valley University, Qena 83523, Egypt.

^8^Department Clinical Pathology, Faculty of Medicine, Tanta University, Tanta, Egypt.

^9^Department of Physical Therapy for Surgery and Burn, Faculty of Physical Therapy, South Valley University, Qena 83523, Egypt.

****Corresponding author***: Prof. Mohammed H. Hassan, Professor of Medical Biochemistry, Faculty of Medicine, South Valley University, Qena 83523, Egypt.

E-mail: [mohammedhosnyhassaan@med.svu.edu.eg](mailto:mohammedhosnyhassaan@med.svu.edu.eg); [Mohammedhosnyhassaan@yahoo.com](mailto:Mohammedhosnyhassaan@yahoo.com)

ORCID: 0000-0003-2698-9438.


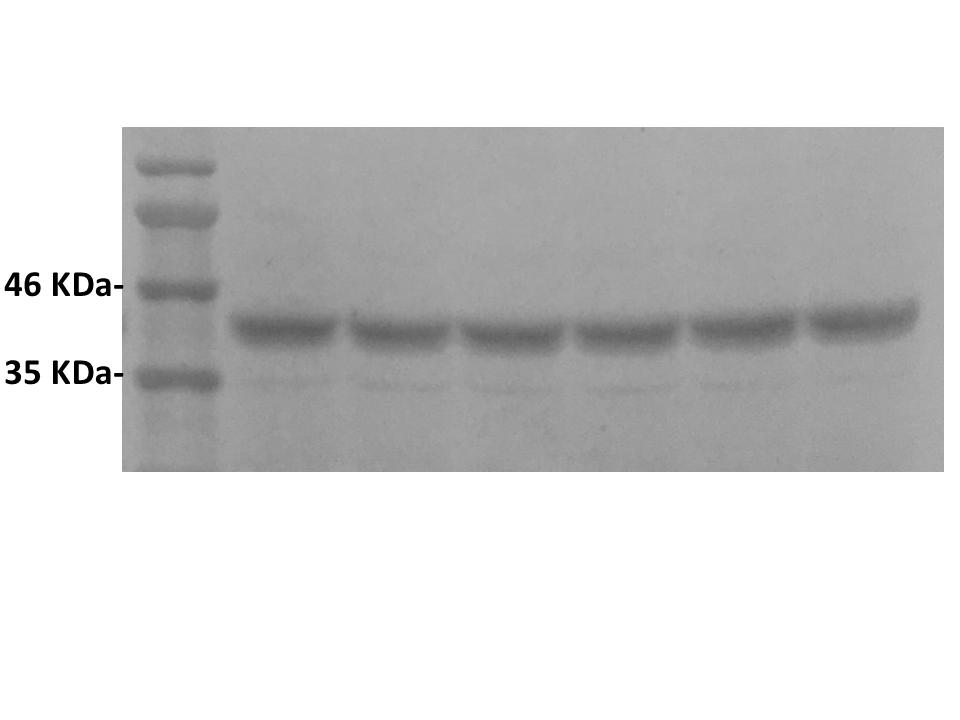

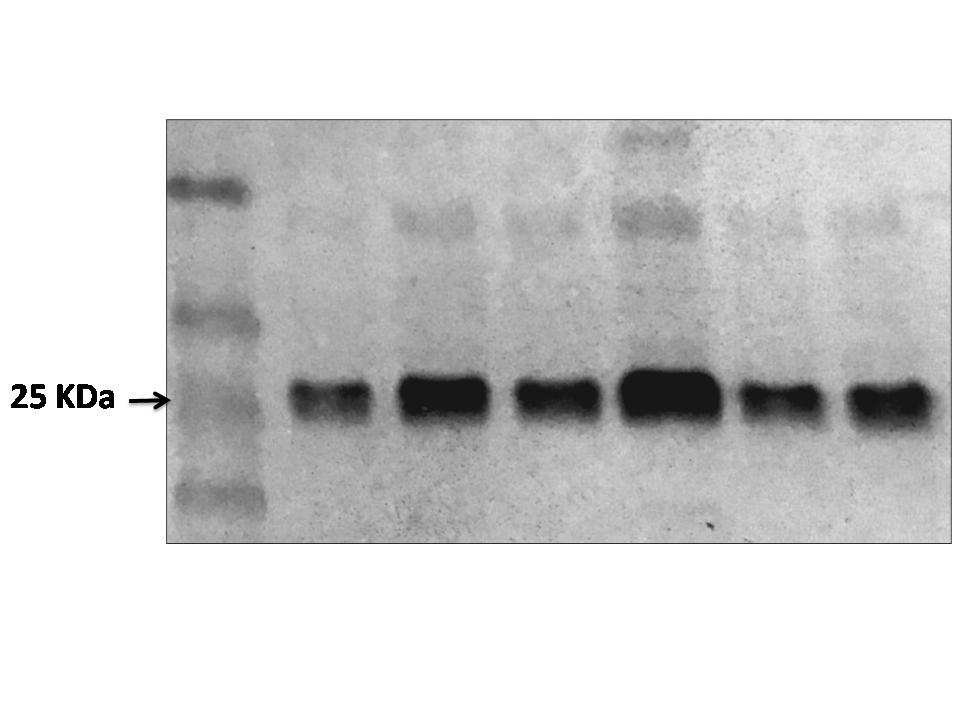

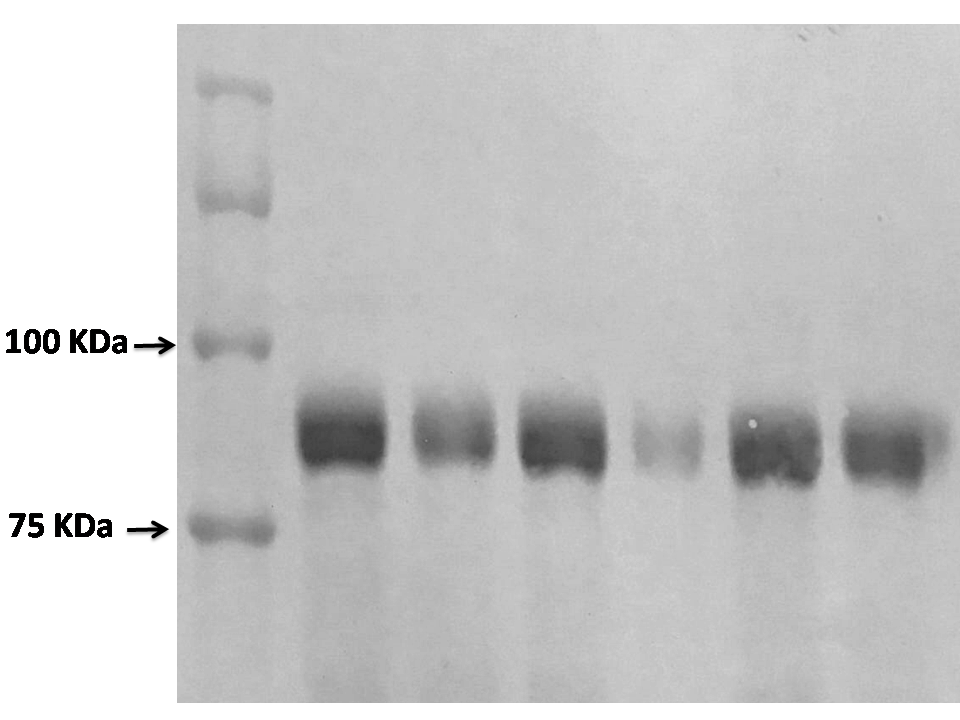

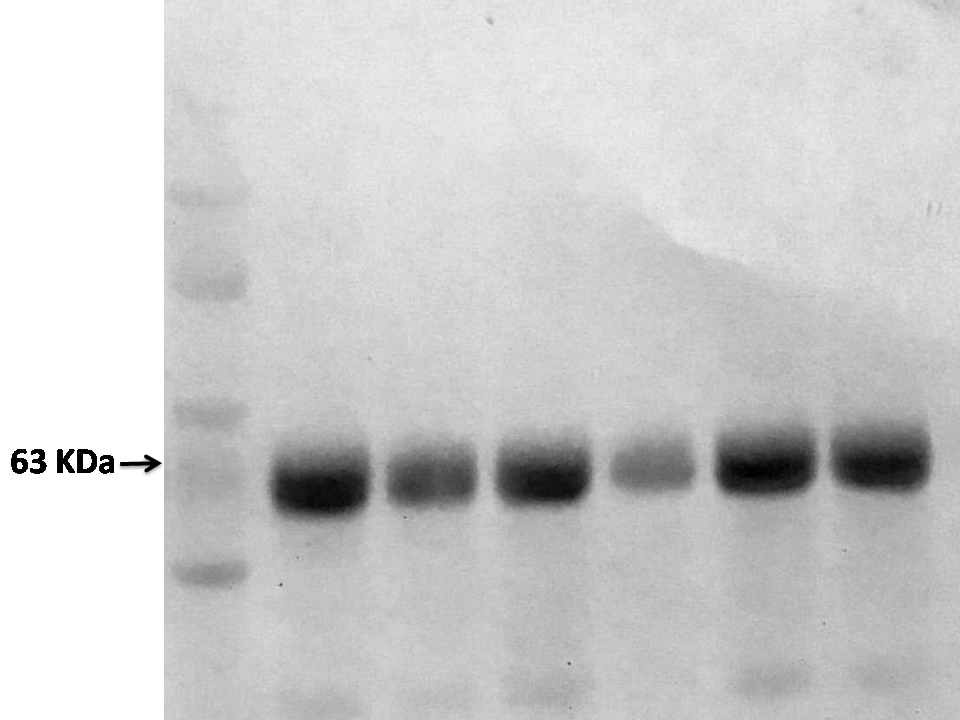


**D**

**C**

**A**

**B**

**Supplementary Fig.1.** Full uncropped and unedited versions of the western blots for MMP-8 (**A**), MMP-9 (**B**), TIMP-1 (**C**), and β-actin (**D**) expression levels in different patients’ groups

| 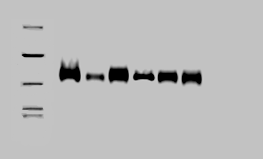  **A** | 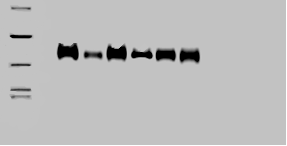  **B** |
| --- | --- |
| 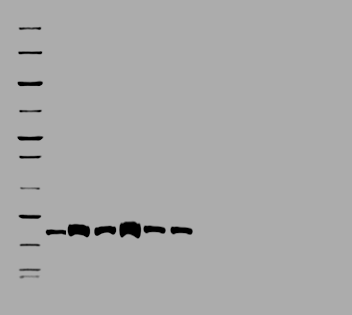  **C** | **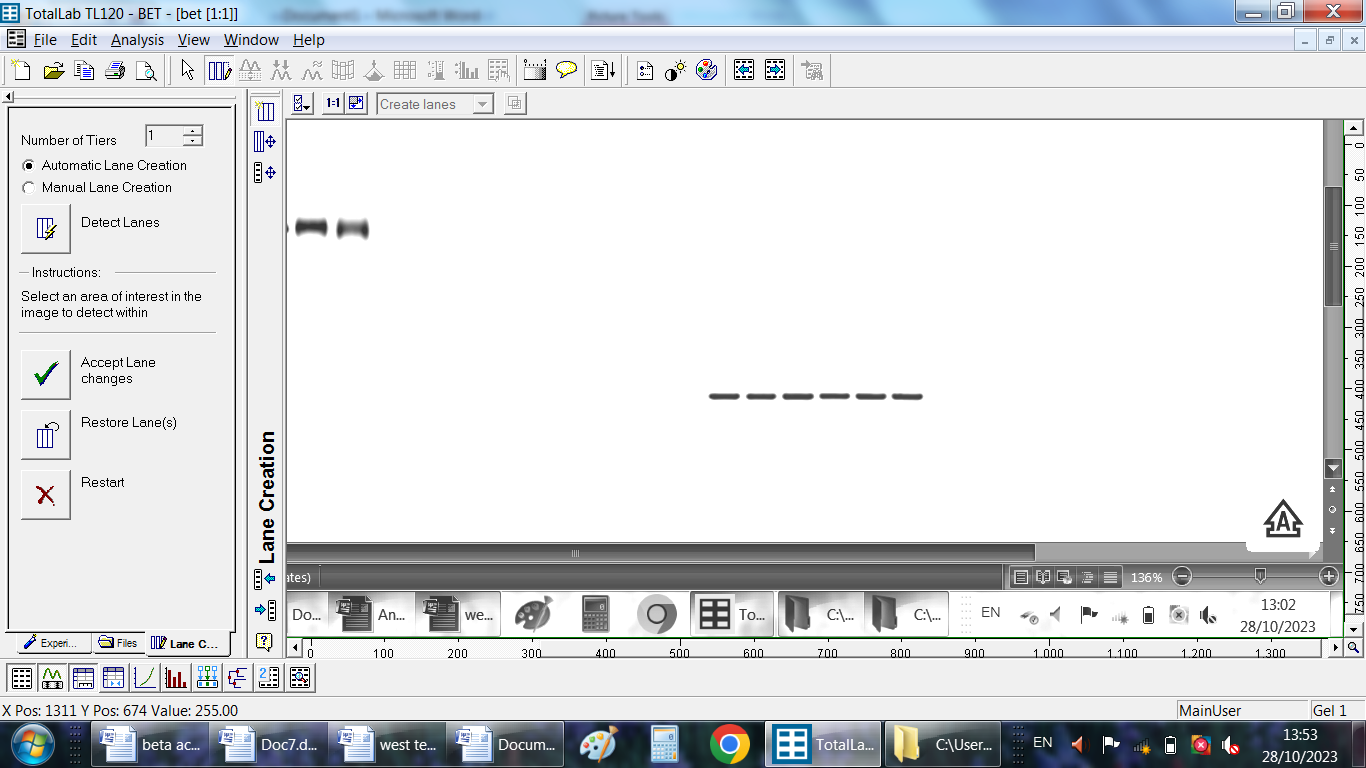**  **D** |

**Supplementary Fig.2.** Full uncropped and unedited versions of the western blots for MMP-8 (**A**), MMP-9 (**B**), TIMP-1 (**C**), and β-actin (**D**) expression levels in different patients’ groups.

| 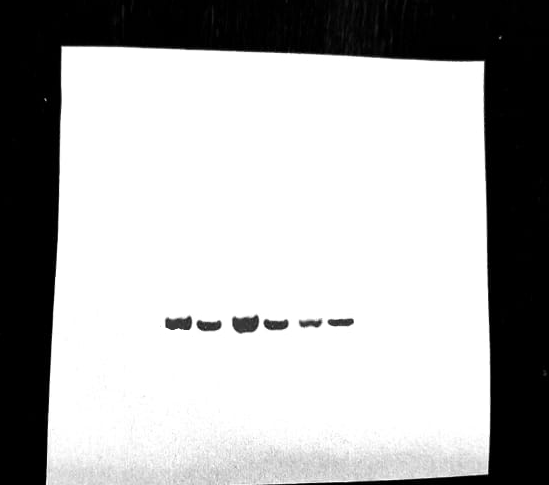  **A** | 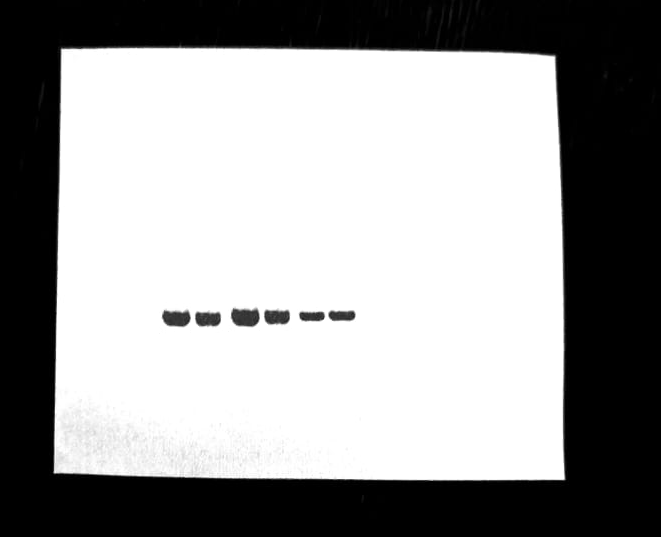  **B** |
| --- | --- |
| 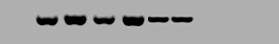  **C** | 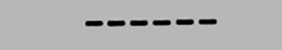  **D** |

**Supplementary Fig.3.** Full uncropped and unedited versions of the western blots for MMP-8 (**A**), MMP-9 (**B**), TIMP-1 (**C**), and β-actin (**D**) expression levels in different patients’ groups.
